# Evaluation of the peak experience scale as a rapid assessment tool for the strength of a psychoactive experience with 5-MeO-DMT

**DOI:** 10.3389/fpsyg.2025.1543640

**Published:** 2025-06-02

**Authors:** Johannes T. Reckweg, Natasha L. Mason, Eef L. Theunissen, Claus B. Svendsen, Theis H. Terwey, Johannes G. Ramaekers

**Affiliations:** ^1^Faculty of Psychology and Neuroscience, Maastricht University, Maastricht, Netherlands; ^2^GH Research, Dublin, Ireland

**Keywords:** psychedelic, psychoactive, mebufotenin, 5-MeO-DMT, questionnaire, peak experience

## Abstract

A three-item Peak Experience Scale (PES) was developed to rapidly evaluate the strength of the psychoactive experience, and to guide the dosing regimen, of the psychedelic 5-methoxy-N,N-dimethyltryptamine (5-MeO-DMT; mebufotenin). This paper aims to compare the PES with a range of established questionnaires designed to evaluate the psychedelic experience. Data were gathered from three separate studies in which a 5-MeO-DMT formulation (GH001) was administered via pulmonary inhalation to healthy volunteers and patients with treatment resistant depression (*N* = 84) as either single doses (0 [placebo], 2, 6, 12, 18 mg) or an incremental individualized dosing regimen (IDR). Apart from the PES, participants also completed the Mystical Experience Questionnaire (MEQ-30), the Challenging Experience Questionnaire (CEQ), the Ego Dissolution Inventory (EDI) and the 5-Dimensional Altered States of Consciousness Rating Scale (5D-ASC). The 5-MeO-DMT formulation produced a significant, dose-related increase in PES ratings, with maximal ratings being achieved after the IDR. A principal component analysis (PCA) of the PES items identified a single primary component explaining 83.5% of the variance. PES items also displayed a strong internal consistency (Cronbach’s *α* = 0.896). A PCA across all questionnaires indicated a strong and unidimensional loading of the PES, MEQ, EDI and the 5D-ASC, suggesting high interrelatedness. Likewise, individual ratings on the PES were highly correlated to those on the PES, MEQ, EDI and the 5D-ASC, but not the CEQ. The PES is concluded to be an effective tool to rapidly assess the strength of the psychedelic experience with 5-MeO-DMT. The PES could prove useful to gain fast insight into the strength of a psychedelic dose in individual patients and potentially guide dose and re-dose selection of rapid-acting psychedelics.

## Introduction

The psychoactive experience (PsE) with a psychedelic is often marked by its phenomenological ineffability and can contain alterations in consciousness that are difficult to define qualitatively and quantitatively. Such alterations include visual and auditory distortions, altered social and emotional processing, increased bliss and a loss of self-identity that can evoke a strong sense of spiritual or mystical meaning (Nichols, 2016).

Subjective questionnaires have been developed by other researchers to capture and quantify the characteristics of the PsE. The most frequently used scales include the 5-Dimensional Altered States of Consciousness Rating Scale [5D-ASC (Studerus et al., 2010)], the Ego-Dissolution Inventory [EDI (Nour et al., 2016)], the Mystical Experience Questionnaire [MEQ-30 (Barrett et al., 2015)], and the Challenging Experience Questionnaire [CEQ (Barrett et al., 2016)]. These subjectively rated scales are used to measure the changes in consciousness elicited by psychedelics, its intensity, and changes in self-referential awareness. Other, less established questionnaires also aim to investigate the influence of the environmental setting on the experience [Setting Questionnaire for the Ayahuasca Experience (Pontual et al., 2021)], as well as potential meaningful insights [Psychological Insight Questionnaire (Davis et al., 2021), Psychological Insight Scale (Peill et al., 2022)] elicited by the psychedelic experience. However, these questionnaires are time-intensive, typically taking 10–20 min to complete, with additional time required to evaluate the results. Currently, no validated questionnaire exists to quickly and accurately assess the intensity and profoundness of a psychedelic experience within a minute. Such a tool would be desirable in a clinical setting, when evaluating the impact of an individual dose and dose-escalations of rapid-acting psychoactives on the psychedelic experience.

The intensity of the psychedelic experience has been demonstrated in a range of studies to be associated with beneficial changes in mental outcome parameters. Observational studies indicated that higher ratings on the EDI and the 5D-ASC, especially its Oceanic Boundlessness (OBN) subscale, after administration of ayahuasca and 5-MeO-DMT were significantly associated with improved satisfaction in life and reductions in symptoms of stress and depression (van Oorsouw et al., 2022; Uthaug et al., 2018; Uthaug et al., 2019). In clinical studies, higher ratings on the MEQ and on the OBN subscale of the 5D-ASC have also been correlated with long-term improvements in symptoms of anxiety following LSD administration (Holze et al., 2023) and long-term antidepressant effects in patients with Bipolar II Disorder after psilocybin administration (Aaronson et al., 2023). Further, higher scores on the MEQ and Hallucinogen Rating Scale (HRS) following ayahuasca administration have been associated with alleviation of symptoms in patients with treatment-resistant depression (Palhano-Fontes et al., 2019). In the same indication, higher scores on the OBN subscale of the 5D-ASC following two psilocybin administrations also predicted positive long-term symptom improvements (Roseman et al., 2018). In patients with symptoms of end-of-life anxiety and depression, a more intense psilocybin experience, as indicated by higher ratings on the MEQ, correlated with greater satisfaction with life, more meaning in life, and lower scores on scales assessing symptoms of depression and anxiety (Griffiths et al., 2016). Additionally, literature reviews support the notion that higher scores on mystical-type effects occasioned by a variety of psychedelics correlated with improvements in depression symptoms and general well-being (Kangaslampi, 2023; Ko et al., 2022), and the intensity of the experience predicts reduction in symptom severity for indications such as substance use disorders, depression, and anxiety (Romeo et al., 2021). Given this association between the strength of the psychedelic effects and potential therapeutic benefits, a quick and reliable measure of the magnitude of psychedelic effects is needed.

Efforts have been made to evaluate the therapeutic efficacy of the short-lasting and rapid-acting psychedelic 5-methoxy-N,N-dimethyltryptamine (5-MeO-DMT; mebufotenin) in patients with treatment-resistant depression (Reckweg et al., 2022; Reckweg et al., 2023). Interestingly, 5-MeO-DMT has proven to have a high propensity to elicit Peak Experiences (PE). Mebufotenin does not induce tachyphylaxis, a term used to describe reduced response to a drug after its administration (Reckweg et al., 2021). This, coupled with the short duration of psychoactive effects of about 5–30 min after inhalation of mebufotenin, allows for same-day redosing, if the desired intensity of effects has not been reached. To support this, a rapid yet reliable evaluation of the dose-induced strength of the psychedelic experience is required. Peak experiences, as described by Maslow (1970), are marked by feelings of transcendence, meaningfulness, and can even occasion feelings of death and rebirth. All these factors can also occur in a psychedelic experience and similarities between Maslow’s description and psychedelic experiences have been drawn (Richards et al., 1977; Cummins and Lyke, 2013). Additionally, the psychoactive experience of 5-MeO-DMT is often distinct to that of other psychedelics. While substances such as psilocybin and LSD induce more characteristic psychedelic effects, an experience with 5-MeO-DMT seems phenomenologically different. Experiences with other classic psychedelics are usually described as more colorful, due to their strong and persistent effects on the visual perception, (Kometer and Vollenweider, 2018) while experiences with mebufotenin are often described as much less visual and more relating to the head space (Palamar and Acosta, 2020). Thus, experiences or memories are much less tangible or distinct with a mebufotenin experience that rather leaves feelings or overarching impressions (Millière et al., 2018). This degree of ‘ineffability’ can make it difficult to properly capture the experience with the previously mentioned questionnaires, which focus more on the stereotypical aspects associated with psychedelic effects. These less tangible effects might be one of the facilitating factors of 5-MeO-DMT in eliciting PEs, as the experience is focused on the actual magnitude of the effects, rather than perceptual changes in the environment, which might be more influential with other classic psychedelics.

A new Peak Experience Scale (PES) was developed to rapidly assess the strength of the psychedelic experience induced by 5-MeO-DMT. In a series of three studies, 5-MeO-DMT was administered as the inhalable formulation GH001. The studies were split into two parts, with participants receiving either a single dose or up to three increasing doses on a single administration day, respectively. The PES comprises three visual analog scale (VAS)-style questions (scored 0–100), evaluating the profoundness and the intensity of the experience, as well as the perceived loss of control of the participant, with an average score of 75 or higher qualifying as a PE. The choice to use a score of ≥75 as a cutoff was based on pragmatism, judging the upper quartile to signal sufficient overall strength of the psychoactive experience. The PES was used after each administration of GH001 to determine if the participant reached a PE, or in the case of the second part, if another administration was needed. It was hypothesized that the PES would be a reliable tool to classify the magnitude of a 5-MeO-DMT experience. The main aim was to determine if the PES could be used in combination with appropriate safety evaluations to guide decisions surrounding the dosing regimen and to evaluate the occurrence of PEs. To determine the sensitivity of the PES in evaluating the occurrence of a PE in relation to the responses on other questionnaires, the 5-Dimensional Altered States of Consciousness Questionnaire (5D-ASC), the Mystical Experience Questionnaire (MEQ), and the Ego-Dissolution Inventory (EDI) were included. The Challenging Experience Questionnaire (CEQ) was included to demonstrate that the PES measures a distinct construct from the CEQ, and to provide evidence that the PES aligns more closely with aspects that are components of a PE such as ego-dissolution or mystical-type experiences.

## Methods

### Studies

A total of three clinical studies were included in the analyses. In each study, a vaporizable 5-MeO-DMT formulation called GH001 was administered, either as single doses (0 mg [placebo], 2 mg, 6 mg, 12 mg, 18 mg) or as part of an individualized dosing regimen (IDR), with up to three increasing doses (6 mg, 12 mg, 18 mg) on a single administration day. The intent was to elucidate which doses would most reliably elicit a PE, as assessed by the PES. In the IDR condition, participants received consecutive doses interspersed by approximately 3 h in Study 1 and 2, and either 1 or 2 h in Study 3. After each dose, the PES was administered to evaluate if a PE was reached. If so, the test day was concluded. If no PE had been reached, the subsequent dose was administered.

Study 1 Reckweg et al. (2021) was a Phase 1 trial in healthy volunteers, investigating the safety and dose-related effects of GH001 in 22 participants. A total of 18 participants received single doses of either 2 mg, 6 mg, 12 mg, or 18 mg, while another four participants went through the IDR condition of up to three doses of 6 mg, 12 mg, and 18 mg in a single testing day (ClinicalTrials.gov Identifier: NCT04640831).

Study 2 Reckweg et al. (2023) was a Phase 1/2 trial in patients with treatment-resistant depression. In the Phase 1 part of the study (*n* = 8) participants received single doses of either 12 mg or 18 mg, with the primary outcome measure being focused on the safety of GH001 in a patient population. In the Phase 2 part (*n* = 8) participants went through the IDR with the main outcome measure being the proportion of remissions on day 7 after administration. A summary of the study can be found in Reckweg et al. (2023) (ClinicalTrials.gov Identifier: NCT04698603).

Study 3 was a Phase 1 study in 46 healthy volunteers, focusing on pharmacokinetics of GH001 in a healthy population. It was a two-part design, with the first part being a double-blind setup. Three groups of 10 participants each first received single doses of either 6 mg, 12 mg, or 18 mg. In each group, two randomized participants received a placebo dose. The second part was an open-label phase with 16 participants going through the IDR condition with two different intervals between dosing (ClinicalTrials.gov Identifier: NCT05163691).

All three studies were approved by the Dutch Central Committee on Research Involving Human Subjects (CCMO) and the relevant institutional review board (IRB), and conducted according to the principles of Good Clinical Practice (GCP) and the code of ethics on human experimentation established by the Declaration of Helsinki (1964) and amended in Fortaleza (2013) (World Medical Association, 2013).

#### The peak experience scale (PES)

The assessment of the intensity of psychedelic effects relied on the proprietary PES, comprised of three questions (“How intense was the experience?,” “To what extent did you lose control?,” “How profound (i.e., deep and significant) was the experience?”), all answered by marking a VAS between 0 and 100, and then averaged to provide the total PES score. A PE was pragmatically defined as the average score greater than or equal to 75. The three items were chosen based on core elements of the psychedelic experience, as observed with other rating scales (Studerus et al., 2010; Nour et al., 2016; Mortaheb et al., 2024; Barsuglia et al., 2018).

#### Ego dissolution inventory (EDI)

The EDI (Nour et al., 2016) is an 8-item self-report scale that assesses the participant’s experience of ego dissolution. Sample items for the scale included the following: “I experienced a dissolution of my “self” or ego” and “I felt at one with the universe.” The purpose of this scale was to acquire a better understanding of the experiences the participant had about ego dissolution during the psychedelic experience. The participant answered the scale with endpoints of either 0 = “No, not more than usual” or 100 = “Yes, I experience this completely/entirely.” The EDI is scored by calculating the mean of all the 8 items (range 0–100). The higher the total score, the stronger the experience of ego dissolution.

#### Mystical experience questionnaire (MEQ)

The MEQ (Barrett et al., 2015) contains 30 items from the previous 43-item version (MEQ-43). The questions of the 30-item MEQ relate to the factors: mystical (including items from the internal unity, external unity, noetic quality, and sacredness scales of the MEQ-43), positive mood, transcendence of time and space, and ineffability (all three of which include items from their respective MEQ-43 scales). Thus, the MEQ retained items from each qualitative subscale in the original MEQ-43, but in a reduced number of dimensions. The main outcome measure was the total score of the MEQ. Responses were indicated on a 6-point scale ranging from “None; not at all” to “Extreme.”

#### Challenging experience questionnaire (CEQ)

The CEQ (Barrett et al., 2016) is comprised of 26 items (e.g., “Sadness,” “Feelings of Despair,” “I felt isolated from everything and everyone”) that make up seven subscales (grief, fear, death, insanity, isolation, physical distress, and paranoia) to provide a degree to which a given psychedelic experience was challenging for the participant. The participant indicated the appropriate response on a 6-point scale, ranging from “None; not at all” to “Extreme.” Response data for each item were divided by the maximum possible response (i.e., 5). The average of all transformed item scores across all items was computed to obtain the CEQ total score.

#### 5-dimensional altered states of consciousness questionnaire (5D-ASC)

The 5D-ASC (Studerus et al., 2010; Dittrich, 1998) is a 94-item self-report scale that assessed the participant’s alterations from normal waking consciousness. The participant was asked to make a vertical mark on a 10 cm VAS scale for each statement in order to rate to what extent the statements applied to their experience in retrospect (e.g., from “No, not more than usually” to “Yes, more than usually”). The 5D-ASC measures 11 subscales: experience of unity, spiritual experience, blissful state, insightfulness, disembodiment, impaired control and cognition, anxiety, complex imagery, elementary imagery, audio-visual synesthesia and changed meaning of percepts. Alternatively, the scores can also be summarized in 5 key dimensions: Oceanic Boundlessness, Anxious Ego Dissolution, Visual Restructuralization, Auditory Alterations, and Reduction of Vigilance.

### Study treatment

GH001 (GH Research, Dublin, Ireland) is an investigational drug product based on a proprietary formulation of synthetic, high purity, GMP pharmaceutical grade 5-MeO-DMT (mebufotenin) for administration via inhalation. GH001 was administered after a standardized vaporization procedure using the Volcano Medic or Volcano Medic 2 Vaporization System (Storz and Bickel, Germany), that was approved for medical use with cannabinoids at the time of the studies in Europe, Australia, and Canada (Gieringer et al., 2004; Abrams et al., 2007; Hazekamp et al., 2006). The device consists of a hot air generator, which facilitates formation of an aerosol from GH001, and a detachable valve balloon from which the aerosol is inhaled by the participant with a single breath. After inhalation, participants were instructed to hold their breath for 10 s before exhaling.

### Procedure

In each study, participants were administered GH001 through inhalation after vaporization. This was followed by a phase of 60 min in Studies 1 and 2, and approximately 30 min in Study 3, in which the participant did not have to complete any scales. The volunteers then completed a range of questionnaires, among them the PES that was used to determine if a PE was reached. For a detailed example of testing day procedures, see Reckweg et al. (2021). The PES was completed from 30 min after administration in Study 3 and from about 90 min after administration in Studies 1 and 2. The other questionnaires on the psychedelic experience were completed immediately after the PES.

### Statistical analyses

The internal consistency of the questions of the PES were evaluated using Cronbach’s alpha. A principal component analysis (PCA) was conducted to measure the interrelationship of the individual items of the PES. An additional PCA was conducted to assess the interrelationship between the constructs covered by the PES, MEQ, CEQ, EDI, and the overall 5D-ASC score as well as its Oceanic Boundlessness subscale. The Kaiser-Meyer-Olkin (KMO) and Bartlett’s tests were performed prior to the PCAs to check for factorial redundancy of the variables. Scree plot criteria were used to decide on the number of factors to include for the components analyses.

ANOVAs with a single factor *Dose* were performed on each of the questionnaires. This factor had 6 levels (Placebo, 2 mg, 6 mg, 12 mg, 18 mg, IDR) for the PES, MEQ, and CEQ, and 5 levels (2 mg, 6 mg, 12 mg, 18 mg, IDR) for the EDI and 5D-ASC. The difference in the number of levels is due to the PES, MEQ, and CEQ being included in Study 3, which included a placebo condition, while the EDI and 5D-ASC were not. Pearson’s correlations were performed between the main outcome measure of the PES and the other questionnaires.

For the IDR condition, only ratings as assessed after the final administration of GH001 was included in the analyses. All analyses and graph formation were performed using IBM SPSS Statistics for Windows, Version 28.0 and GraphPad Prism Version 9.4.1.

## Results

### Missing data

Data for the MEQ was excluded due to missing data points for two participants of Study 3.

### Study demographics

A summary of participant demographics can be found in Table 1.

**Table 1:** Demographic data for each study.

Study	Gender	Age, median (range)
Study 1	13 male (59.1%), 9 female	28.5, (18–42)
Study 2	9 male (56.3%), 7 female	29.5 (21–51)
Study 3	28 male (60.9%), 18 female	27.5 (19–59)

### Subjective drug effects

**Figure 1:**
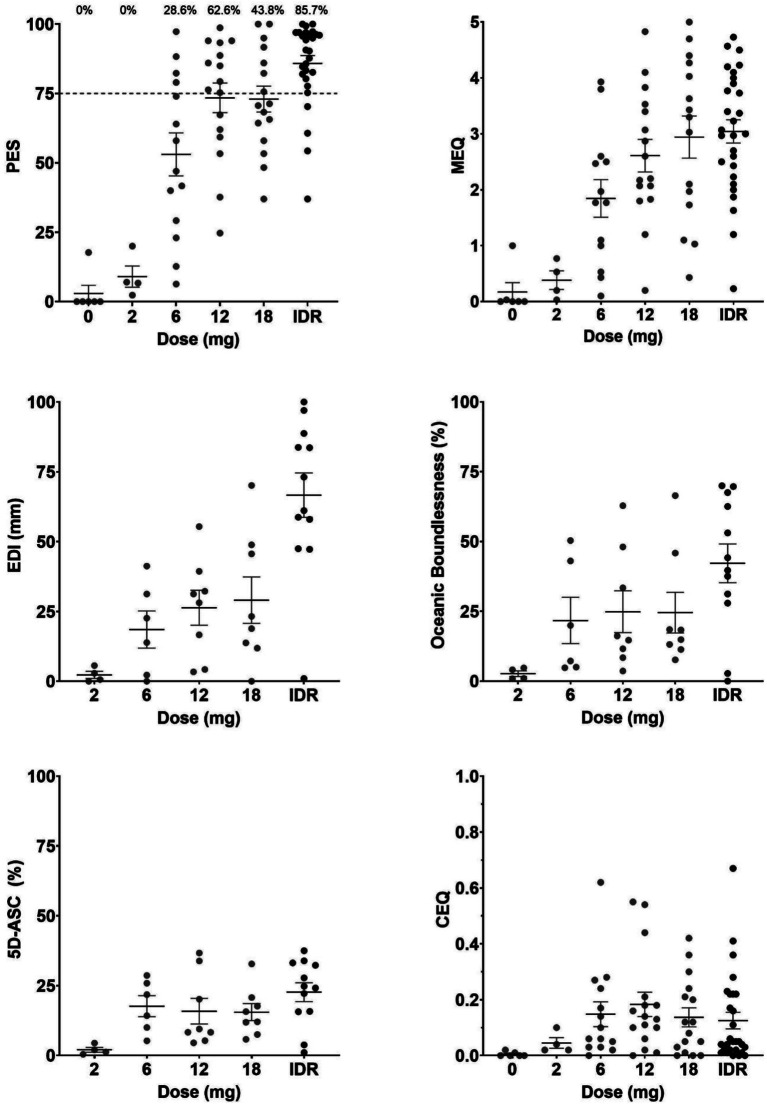
Means (SEM) and individual ratings of the psychedelic experience per dose level for the PES, EDI, MEQ, CEQ, 5D-ASC, and the oceanic boundlessness subscale of the 5D-ASC. Percentages above the PES indicate the proportion of participants achieving a PE per dose.

Mean with standard error (SEM) and individual ratings for the total outcome of each questionnaire, as well as the Oceanic Boundlessness subscale of the 5D-ASC, are shown in Figure 1. ANOVAs indicated a significant effect of *Dose* of 5-MeO-DMT on ratings of the PES (*F*_5,78_ = 27.271, *p* < 0.001, *η*_p_^2^ = 0.636), EDI (*F*_4,33_ = 9.836, *p* < 0.001, *η*_p_^2^ = 0.544), MEQ (*F*_5,76_ = 10.024, *p* < 0.001, *η*_p_^2^ = 0.397), 5D-ASC (*F*_4,33_ = 2.984, *p* = 0.033, *η*_p_^2^ = 0.266), and its subscales Oceanic Boundlessness (*F*_4,33_ = 3.048, *p* = 0.03, *η*_p_^2^ = 0.27) and Reduction of Vigilance (*F*_4,33_ = 3.096, *p* = 0.029, *η*_p_^2^ = 0.273). There was no significant effect of *Dose* on the CEQ (*F*_5,78_ = 1.548, *p* = 0.185, *η*_p_^2^ = 0.09), or the 5D-ASC subscales Anxious ego dissolution (*F*_4,33_ = 1.33, *p* = 0.279, *η*_p_^2^ = 0.139), Visual restructuralization (*F*_4,33_ = 1.625, *p* = 0.191, *η*_p_^2^ = 0.165), or Auditory alterations (*F*_4,33_ = 1.149, *p* = 0.351, *η*_p_^2^ = 0.122).

### Characteristics of the PES

#### Principal component analyses

A PCA was conducted to assess the association between PES items. The KMO test revealed a shared variance between the variables of 0.72. Bartlett’s test for sphericity was significant (*χ*^2^ = 161.357, *p* < 0.001). These results indicate the suitability of the data for dimensional reduction with a PCA. The PCA identified a major primary component that explained 83.5% of the variance (eigenvalue = 2.506). The subsequent components had eigenvalues below 0.5 and were not included. The three items on the PES, i.e., Intensity of the experience, Loss of Control, and Profoundness, loaded on the component with a factor 0.944, 0.894, and 0.902, respectively.

Another PCA was conducted to assess the interrelationship between the main outcome variables of the questionnaires compared to each other (i.e., PES, MEQ, EDI, 5D-ASC, CEQ). The KMO test indicated a shared variance between the variables of 72.6% while the Bartlett’s test for sphericity achieved significance (*χ*^2^ = 142.95, *p* < 0.001). The PCA subsequently identified two major explanatory components from the questionnaire dataset. A first component explained 68.4% of the variance (eigenvalue of 3.42) and was strongly associated with the main outcome parameter of all questionnaires, except for the CEQ. A second component explained 19.4% of the variance (eigenvalue = 0.97) and was primarily associated with CEQ ratings. Together, these components explained a cumulative 87.8% of the variance in the questionnaire ratings. Subsequent components had eigenvalues below 0.5 and were not included.

The component matrix, indicating how much each factor (i.e., questionnaire) loads onto each of the included components, is shown in Table 2.

**Table 2:** Component matrix for the PCA indicating the extent to which each factors load onto the two components.

Questionnaire	Component	
	1	2
PES	0.841	0.018
EDI	0.909	−0.211
MEQ	0.962	−0.120
CEQ	0.324	0.938
5D-ASC (%)	0.903	0.183
Oceanic boundless (%)	0.926	−0.192

#### Internal consistency

Cronbach’s alpha indicated an internal consistency of 0.896 for the three questions of the PES, suggesting a high reliability of the items.

#### Correlations of the PES with other scales

**Figure 2:**
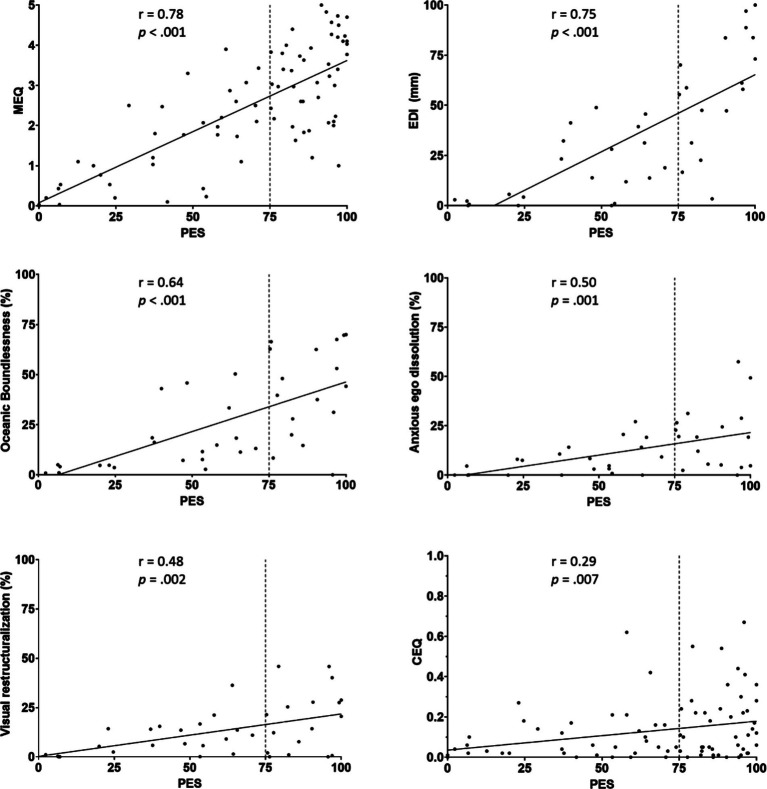
Scatterplots and Pearson correlation coefficient indicating the relation between PES and other measures of the psychedelic experience (CEQ, MEQ, EDI), as well as the 5D-ASC subscale of Oceanic Boundlessness, Anxious Ego Dissolution, and Visual Restructuralization.

Pearson’s correlations indicated moderate to strong positive correlations between the PES, the EDI, the MEQ, and the 5D-ASC subscales Oceanic Boundlessness, Anxious Ego Dissolution, and Visual Restructuralization. The correlation between the PES and the CEQ was very weak. A graphic representation of the significant correlations between PES and the other questionnaires can be seen in Figure 2. While most questionnaires and subscales correlated significantly and strongly with the PES, the 5D-ASC subscales of Auditory Alterations (*r*(38) = 0.174, *p* = 0.297) and Reduction of Vigilance (*r*(38) = 0.132, *p* = 0.43) did not. The correlations between the PES and other scales were not corrected for multiple comparisons. However, all significant correlations would have survived a Bonferroni correction.

## Discussion

Data from three studies that administered the PES following administration of a vaporized 5-MeO-DMT (mebufotenin) formulation (GH001) were pooled to assess the value of the PES as a determinant of the strength of a psychoactive experience with 5-MeO-DMT. To this end, PES scores were evaluated for their assessment of the dose response and related to outcomes on established questionnaires in the current literature, namely the 5D-ASC, MEQ, CEQ, and EDI. Of these scales, the PES showed the strongest response to effects of 5-MeO-DMT as assessed by ANOVAs (*F*_5,78_ = 27.271, *p* < 0.001, *η*_p_^2^ = 0.636). The PES also showed correlations with the MEQ, EDI, and the Oceanic Boundlessness, Anxious Ego Dissolution, and Visual Restructuralization subscales of the 5D-ASC. Evaluations of the internal consistency of the PES suggested a high reliability of its items, with a PCA indicating that the three items on the PES explain a total of 83.53% of the variance of the scores. An additional component analysis with the other scales indicated that 68.4% of the variance was explained mostly by the PES, MEQ, EDI, and 5D-ASC, while the CEQ almost exclusively loaded on a second component, explaining an additional 19.4% of the variance. Together, these results support claims of the PES as a reliable and highly consistent tool to swiftly determine the strength of a 5-MeO-DMT experience. This supports the PES as a tool to guide dosing decisions and to determine the occurrence of a PE.

Consistent with the hypothesis that the PES would reliably depict the magnitude of effects following administration of different doses of 5-MeO-DMT, ANOVAs highlighted the largest effect sizes of *Dose* on the various scales. While the MEQ (*η*^2^ = 0.397), EDI (*η*^2^ = 0.544), and 5D-ASC (*η*^2^ = 0.266) also showed significant changes, effect sizes indicated this effect to be most pronounced for the PES (*η*^2^ = 0.636), thus, indicating a clear dose response for the PES. Additionally, compared to single doses, the IDR condition seems to be the most effective and reliable dosing schedule to cause a PE with GH001, with almost 86% of participants reaching a PE. Overall, this suggests the PES to be effective in depicting the strength of a psychedelic experience, especially so in a regimen with multiple doses of GH001 within a single day. The IDR with GH001, in conjunction with the PES, provides an intriguing opportunity in pharmacology, by – in combination with safety measures – tailoring the required dose for a patient within a few hours. This is expected to reduce the frequency of under- or overdosing, thus reducing risks and strain on the patients.

The PES was designed to get an impression of the magnitude of effects of GH001. Hence, a three-item inventory was chosen to avoid overlap with other facets of the psychedelic experience, focusing on aspects relating to the strength of the experience. As indicated by a PCA, the three items of the PES indeed loaded very strongly on a single primary component, which explained 83.5% of the variance of scores. All other components yielded eigenvalues below 0.5, confirming the PES to be a univariate outcome measure. This is further supported by another PCA on the interrelationship with other scales, indicating strong component loadings for the PES, MEQ, EDI, and 5D-ASC on the same primary principal component. This component could relate to feelings of ego dissolution and mystical-type experiences or transformative experiences, all of which have been related to increased scores on the outcome variables (Reckweg et al., 2021). Together, these PCAs point toward a single outcome variable of the PES that is consistent with other questionnaires assessing characteristics related to the strength of a psychedelic experience. To underline this, the PES showed a very strong internal consistency, as indicated by the magnitude of the Cronbach’s alpha. This supports the PES as a streamlined and targeted tool to quantify the strength of an experience with short-lasting psychedelics such as 5-MeO-DMT.

In a direct comparison with the other questionnaires, the PES showed strong correlations with responses on the MEQ, EDI, and moderately strong correlations with the total 5D-ASC and its Oceanic Boundless, Anxious Ego Dissolution, and Visual Restructuralization subscales. While the correlation of the PES with the CEQ was significant, it remained rather weak (*r* = 0.29, *p* = 0.007). Further, while the other questionnaires in the PCA loaded strongly on a component relating to mystical-type experiences, the CEQ contributed heavily to its own component, with the other questionnaires contributing quite little. These results suggest, as expected, the CEQ to be measuring a distinct construct more relating to the difficult aspects of the psychedelic experience (e.g., fear, distress, or feelings of paranoia) (Barrett et al., 2016), while questionnaires such as the MEQ, EDI, and now also PES, seem to be characterizing aspects of transformative experiences.

Consequently, while there seems to be an overlap between the established questionnaires in psychedelic research, the PES appears to add further flexibility in assessing the psychedelic experience, beyond what established tools offer. While the PES does not capture the granular detail of a psychedelic experience, our results suggest that it is able to indicate the overarching strength or magnitude of such an experience. This strength, which as mentioned, can potentially be a predictor for the therapeutic potential of psychedelic compounds in the clinical setting, seems to be reliably assessed by the PES. While the 5D-ASC also employs VAS scales with similar themes as the PES, the ease of administration and short assessment time of the PES make it stand out as a rapid and resourceful tool for clinical applications. Additionally, the added dimension of using the ≥75 average on the three items of the PES as a cutoff to guide the dosing schedule, differentiates the PES from other validated scales.

While there seems to be merit to the PES to assess the strength and seemingly more ‘desirable’ aspects of a psychedelic experience in terms of a potential therapeutic outcome, it is limited in capturing the more undesirable effects. As effects such as paranoia, fear, or anxiety can also occur during the acute phase with all classic psychedelics, questionnaires that capture these characteristics, such as the CEQ, still provide value. Due to the brief nature of the PES, the 5D-ASC, EDI, and MEQ thus provide a more nuanced depiction of the subjective effects of a psychedelic. Further, the PES has only been used in studies with 5-MeO-DMT, so generalizability to other psychedelics has not yet been established. Future studies employing the use of other compounds or even alternative inductors of altered states of consciousness such as breath work, could provide more information if the PES can also capture the strength of those experiences. Another limitation is the currently unestablished ability of the PES to predict a therapeutic outcome of psychedelic experience. If the strength of a psychedelic experience really can predict therapeutic outcomes, achieving a PE, as determined by the PES, should also strongly correlate with alleviations in depressive symptoms. Although Study 2 has used the PES in a small sample to guide the dosing schedule, and found PEs to be a strong predictor of therapeutic results in patients with TRD, (Reckweg et al., 2023) larger samples are needed to evaluate test–retest reliability, discriminative validity, and predictive validity of the PES in future studies. Lastly, while the PES has a strong association with the MEQ, which has been deemed a predictor for therapeutic effects (Ko et al., 2022; Yaden and Griffiths, 2020), increasing focus is being given to other aspects of the psychedelic experience. Factors such as psychological insight or emotional breakthroughs have also been implicated in predicting positive effects in patient populations (Kangaslampi, 2023), and it remains to be seen if the PES is also associated with those effects.

In conclusion, the PES has been developed as a tool to rapidly quantify the strength of a psychedelic experience with GH001 and can determine the occurrence of PEs. The PES might be utilized to rapidly gain insight into the strength of a psychedelic dose in individual patients and potentially guide dose and re-dose selection of pharmacological courses employing rapid-acting psychedelics, allowing for same-day re-dosing.

## Data Availability

The datasets presented in this article are not readily available because data are proprietary to GH Research. The datasets presented in this article are not readily available to protect proprietary information. Requests to access the datasets should be directed to clinicaltrials@ghres.com. Any further enquiries can be directed to the corresponding author/s.
